# Induction therapy in kidney transplant recipients: Description of the practices according to the calendar period from the French multicentric DIVAT cohort

**DOI:** 10.1371/journal.pone.0240929

**Published:** 2020-10-22

**Authors:** Julie Boucquemont, Yohann Foucher, Christophe Masset, Christophe Legendre, Anne Scemla, Fanny Buron, Emmanuel Morelon, Valérie Garrigue, Vincent Pernin, Laetitia Albano, Antoine Sicard, Sophie Girerd, Marc Ladrière, Magali Giral, Jacques Dantal, for the DIVAT consortium

**Affiliations:** 1 INSERM UMR 1246—SPHERE, Nantes University, Tours University, Nantes, France; 2 Centre Hospitalier Universitaire de Nantes, Nantes, France; 3 CRTI UMR 1064, Inserm, Université de Nantes; ITUN, CHU Nantes; RTRS « Centaure », Nantes, France; 4 Kidney Transplant Center, Necker University Hospital, APHP, RTRS « Centaure », Paris Descartes and Sorbonne Paris Cité Universities, Paris, France; 5 Nephrology, Transplantation and Clinical Immunology Department, RTRS « Centaure », Edouard Herriot University Hospital, Hospices Civils, Lyon, France; 6 Nephrology, Dialysis and Transplantation Department, Lapeyronie University Hospital, Montpellier, France; 7 Department of Nephrology and Renal Transplantation, Hospital Pasteur, Nice, France; 8 Renal Transplantation Department, Brabois University Hospital, Nancy, France; 9 Centre d’Investigation Clinique en Biothérapie, Nantes, France; Imperial College Healthcare NHS Trust, UNITED KINGDOM

## Abstract

**Background:**

There is extensive literature with comparisons between Anti-Thymocyte Globulin (ATG) and Basiliximab (BSX) as induction therapy in kidney transplant recipients. The purpose of our benchmarking study was to describe the consequences in terms of practices in 6 transplantation centers of a French prospective cohort.

**Methods:**

We included adult patients who received a first or second kidney graft between 2013 and 2019 (n = 4157). We used logistic regressions to identify characteristics associated with the use of ATG or BSX.

**Results:**

Use of ATG between the centers ranged from 41% to 75%. We observed different factors associated with the treatment decision. Compared to a first transplant, performing a second graft was the only factor significantly associated with the choice of ATG in all centers. The AUC ranged from 0.67 to 0.91, indicating that the centers seemed to define their own rules. As a result, for patients with the same low immunological risk, the probability of receiving ATG varied from 7% to 36%. We stratified the analyses according to two periods, from 2013 to 2015 and from 2016 to 2019. A similar heterogeneity was observed, and in some cases ATG indications between the centers were inverted.

**Conclusions:**

The heterogeneity of induction therapy practices did not decrease in France, even if the reated literature is prolific. This illustrates the necessity to improve the literature by using meta-analyses of recent studies stratified by graft and patient profiles.

## Introduction

The two main induction therapies used in kidney transplantation are anti-thymocyte globulin (ATG) and Basiliximab (BSX). ATG (Thymoglobulin® or Grafalon Fresenius®) is a polyclonal antibody primarily targeting T-cells but also other immune and non-immune cells [1, 2]. BSX (Simulect®) is a chimeric mouse-human monoclonal antibody that targets the α chain (CD25) of the T-cell IL-2 receptor.

Whilst ATG is known to provide better outcomes for high immunological risk patients [3–5], so far there is no evidence of an effect on graft survival [6, 7]. ATG can also be used to avoid steroid use and to delay the use of calcineurin inhibitors (CNI) without increasing rejection rates [8, 9]. It may also prevent delayed graft function (DGF), which is known to increase allograft immunogenicity and the risk of acute rejection [10–13]. ATG is therefore recommended after a long cold ischemia time (CIT) or for recipients receiving an expanded criteria donor graft (ECD, including donors after cardiac death), two possible risk factors of DGF.

ATG-related adverse events are also well-documented. It can induce cytokine release syndrome and serum sickness [2, 14]. Whilst it is also associated with higher rates of CMV infection [11, 15], other issues such as the risk of cancer or diabetes remain uncertain [11, 16–18]. Consequently, for patients at low risk of acute rejection or DGF, BSX, or even no induction therapy, might be more relevant [19].

The theoretical risk-benefit related to the choice between ATG and BSX appears to be based on recommendations from moderate quality evidence and guidelines based on studies mostly conducted before 2013 [19–21]. These recommendations which are based on the efficacy in preventing graft rejection and DGF must also be counterbalanced against the risk of serious adverse events, as described above. For instance, even though elderly recipients are more likely to receive marginal grafts, this population is also more sensitive to ATG-related side effects due to the reduction in immune system function that occurs with age. Few studies have investigated the factors associated with the choice of treatment in transplantation [22–24]. In general, these show that variations in the prescribed treatment was more reflective of center pratices than patients’ profiles. However, these studies were conducted using US registry data and/or pharmacy fill records for patients transplanted before 2015. Furthermore, only the Dharnidharka et al study [22] investigated induction therapy, whilst the two others focused on immunosuppression regimens. To our knowledge, no recent study of a European cohort exists. The purpose of our benchmarking study was to describe the current prescription patterns for induction therapy using ATG compared to BSX in each transplantation center in a French prospective cohort, and to determine whether these have changed over time.

## Materials and methods

### Patients

We extracted data from the French DIVAT cohort (www.divat.fr) of kidney transplant recipients in Lyon, Montpellier, Nancy, Nantes, Nice, and Paris Necker. Data from the DIVAT cohort is available free of charge to academic researchers after a request to its scientific council. The procedure is explained at this address: http://www.divat.fr/access-to-data. The “Comité National Informatique et Liberté” approved the study (CNIL number for the cohort: #891735) and written informed consent was obtained from the participants.

We randomly assigned a letter for each center (A to F). In this study, we focused on first and second kidney transplantations performed between January 2013 and December 2019 in adults, receiving a transplant from a living donor or deceased donor and having received either ATG or BSX as induction therapy. Note that we had no data for 2018 and 2019 for center D, and no data for 2017 to 2019 for center F because of a delay in the data collection. We excluded donors after cardiac death and multiple organ transplants. We also excluded grafts with missing data for anti-HLA class I or II immunization before transplantation due to their importance in the choice of induction therapy. This represented between 0.6% and 10.0% of the patients in each center. The characteristics of included and excluded recipients are described in S1 Table.

### Available data

We considered the following parameters: recipient age, recipient gender, recipient body mass index (BMI), diabetes, cardiovascular and cancer history, recipient cytomegalovirus (CMV) serostatus, CIT, renal replacement therapy, donor age, donor gender, donor type, donor CMV status, donor and recipient Epstein-Barr virus (EBV) serostatus, graft year, transplantation rank, last donor creatinemia level, number of HLA incompatibilities (A+B+DR), and anti-HLA class I and II immunization. For this last parameter, all centers used Luminex technology for anti-HLA antibody determination, except center D where lymphocytotoxic antibody determination was used for half of the transplantations. Flow cytometry MFI thresholds used to define positivity were: 2000 in center A, 1000 in centers C and D, and 500 in centers B, E, and F.

### Statistical analyses

We used two-sided ANOVA, Student’s t-tests, Chi-square, or Fisher statistics for the cohort description. We fitted six logistic regressions to describe the determinants of the induction in each center. We considered all of the significant parameters differentially expressed between the ATG and the BSX groups (p<0.20). To obtain the same set of determinants for each center, we progressively removed the non-significant parameters for all centers (p>0.05). For each resulting model, we estimated the receiver operating characteristic (ROC) curves and the corresponding areas under the curve (AUC). The confidence intervals were obtained by non-parametric bootstrapping (1000 iterations). To investigate potential recent changes in characteristics associated with the choice of induction therapy, we stratified the analyses on two periods: from 2013 to 2015 and from 2016 to 2019. All analyses were performed using R version 3.6.

## Results

### Cohort description

As shown in Table 1, 2195 of the 4157 included kidney transplant recipients (52.8%) were treated by ATG, and this ranged from 41% to 75% between the centers. One can note that the immunization profile of patients also varied between centers. The proportion of pre-transplantation anti-HLA immunized patients was higher in center E (class I: 81.9%, class II: 82.9%) but lower in center B (class I: 19.0%, class II: 20.8%) and center F (class I: 22.6%, class II: 21.3%).

**Table 1:** Cohort characteristics according to the six centers.

	NA	Overall (n = 4157)		A (n = 812)		B (n = 809)		C (n = 817)		D (n = 360)		E (n = 1045)		F (n = 314)		p-value
**Recipient characteristics**																
Recipient age (years)	0	53.1	(14.8)	55.2	(14.9)	51.6	(14.7)	54.2	(13.9)	53.0	(15.5)	50.6	(14.8)	56.9	(14.4)	<0.001
Male recipient	0	2670	(64.2)	501	(61.7)	492	(60.8)	546	(66.8)	247	(68.6)	659	(63.1)	225	(71.7)	0.001
Recipient BMI ≥ 30 kg/m^2^	24	649	(15.7)	126	(15.5)	136	(16.8)	118	(14.8)	72	(20.0)	142	(13.6)	55	(17.5)	0.063
Diabetes history	0	787	(18.9)	151	(18.6)	144	(17.8)	151	(18.5)	72	(20.0)	193	(18.5)	76	(24.2)	0.225
Cardiovascular history (*)	0	1584	(38.1)	373	(45.9)	235	(29.0)	303	(37.1)	210	(58.3)	374	(35.8)	89	(28.3)	<0.001
Cancer history	0	545	(13.1)	160	(19.7)	72	(8.9)	100	(12.2)	41	(11.4)	130	(12.4)	42	(13.4)	<0.001
CMV R+	12	2605	(62.8)	391	(48.2)	542	(67.1)	527	(64.9)	212	(58.9)	715	(68.8)	218	(69.4)	<0.001
Detectable anti-HLA class I	0	1985	(47.8)	308	(37.9)	154	(19.0)	474	(58.0)	122	(33.9)	856	(81.9)	71	(22.6)	<0.001
Detectable anti-HLA class II	0	1908	(45.9)	259	(31.9)	168	(20.8)	442	(54.1)	106	(29.4)	866	(82.9)	67	(21.3)	<0.001
Renal replacement therapy	15															<0.001
Preemptive transplant		717	(17.3)	173	(21.3)	105	(13.0)	121	(15.0)	46	(12.8)	207	(19.8)	65	(20.8)	
Peritoneal dialysis		395	(9.5)	99	(12.2)	96	(11.9)	68	(8.4)	41	(11.4)	64	(6.1)	27	(8.6)	
Hemodialysis		3030	(73.2)	540	(66.5)	608	(75.2)	616	(76.5)	273	(75.8)	772	(74.0)	221	(70.6)	
**Donor characteristics**																
Donor age (years)	21	55.3	(16.5)	57.4	(16.0)	53.7	(17.7)	56.0	(16.5)	53.3	(16.6)	53.9	(15.7)	59.5	(15.4)	<0.001
Male donor	3	2178	(52.4)	438	(53.9)	452	(55.9)	430	(52.7)	176	(48.9)	530	(50.8)	152	(48.6)	0.092
Living donor	0	904	(21.7)	177	(21.8)	125	(15.5)	130	(15.9)	103	(28.6)	334	(32.0)	35	(11.1)	<0.001
CMV D+	0	2347	(56.5)	372	(45.8)	463	(57.2)	495	(60.6)	202	(56.1)	621	(59.4)	194	(61.8)	<0.001
EBV mismatch (+/-)	10	137	(3.3)	34	(4.2)	26	(3.2)	12	(1.5)	10	(2.8)	52	(5.0)	3	(1.0)	<0.001
**Graft characteristics**																
Year	0															<0.001
2013 to 2015		1839	(44.2)	340	(41.9)	311	(38.4)	327	(40.0)	187	(51.9)	441	(42.2)	233	(74.2)	
2016–2017		1294	(31.1)	258	(31.8)	260	(32.1)	226	(27.7)	173	(48.1)	296	(28.3)	81	(25.8)	
2018–2019		1024	(24.6)	214	(26.4)	238	(29.4)	264	(32.3)	0	(0.0)	308	(29.5)	0	(0.0)	
Re-transplantation	0	598	(14.4)	149	(18.3)	100	(12.4)	110	(13.5)	43	(11.9)	158	(15.1)	38	(12.1)	0.004
Last donor creat. ≥ 132.6 μmol/L	12	431	(10.4)	87	(10.7)	80	(9.9)	88	(10.8)	42	(11.7)	85	(8.2)	49	(15.7)	0.007
HLA incompatibilities > 4	55	717	(17.5)	186	(22.9)	124	(15.4)	119	(15.5)	56	(15.6)	183	(17.5)	49	(15.6)	<0.001
Cold ischemia time (hours)	43	13.7	(8.4)	12.0	(7.5)	12.0	(6.6)	16.0	(7.9)	13.4	(7.8)	14.1	(10.6)	15.8	(7.1)	<0.001
ATG depleting induction therapy	0	2195	(52.8)	468	(57.6)	360	(44.5)	611	(74.8)	185	(51.4)	428	(41.0)	143	(45.5)	<0.001

Abbreviations: ATG, anti-thymocyte globulin; BMI, body mass index; CMV, cytomegalovirus; CMV R+, CMV seropositive recipient; CMV D+, CMV seropositive donor; EBV, Epstein-Barr virus; EBV D+R-, EBV seronegative recipient from EBV-seropositive donor; HLA, human leucocyte antigen; NA, number of missing values. Continuous characteristics are presented as means (standard deviation). The qualitative values are presented as the effective (n) modality followed by its percentage. (*) Excluding hypertension. (+/-) EBV positive in the donor and negative in the recipient.

**Fig 1:**
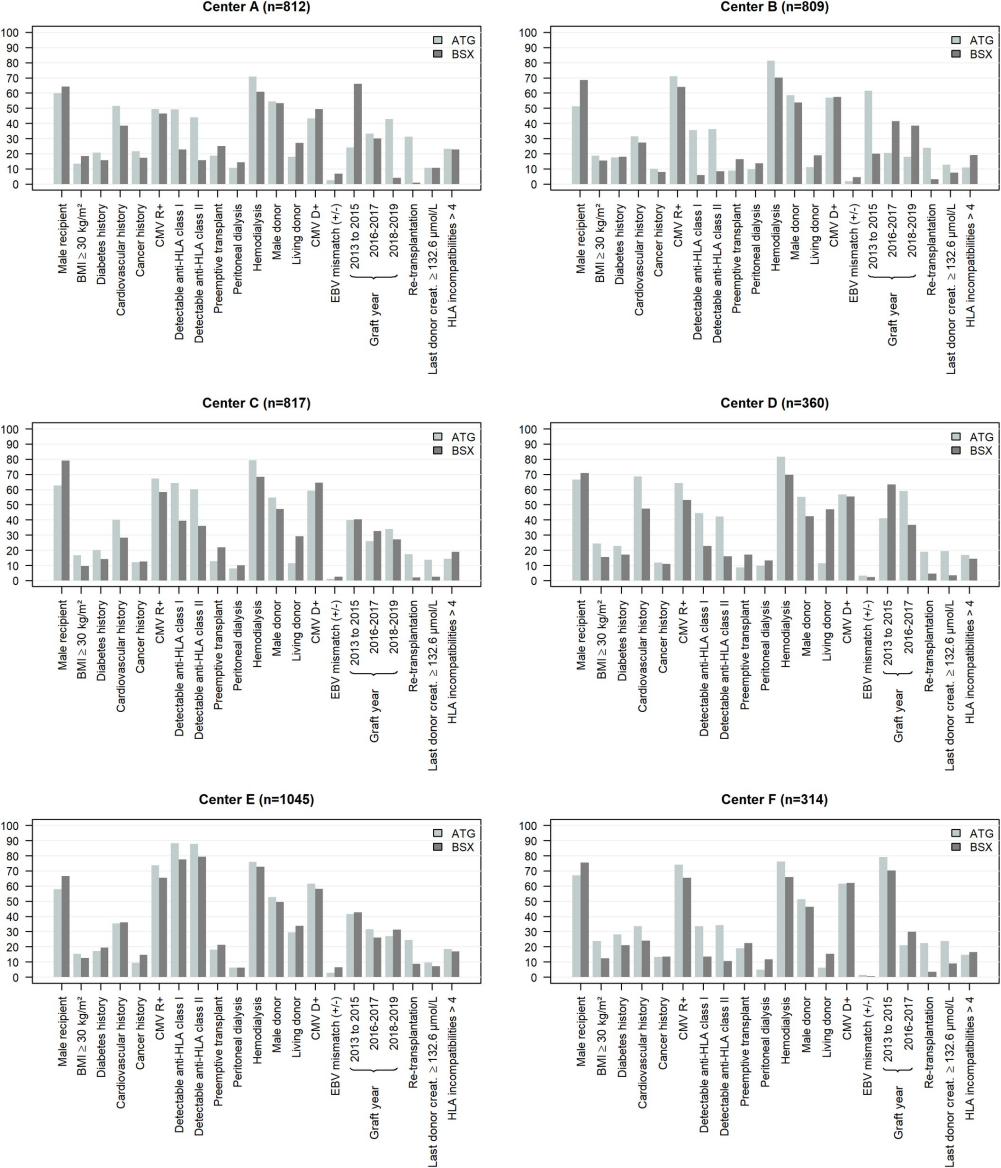
Description of the characteristics of kidney transplant recipients according to induction therapy and center (n = 4157).

Table 2 presents the patient characteristics according to induction therapy in the whole cohort. Fig 1 and S2 to S7 Tables shows the data for each center. In the whole cohort, we observed that women more likely received ATG (40.1%) than BSX (30.9%). Also, ATG was prescribed more often for patients with pre-transplantation anti-HLA class I (57.4%) or II (54.9%) and re-transplantations (23.2%) compared to BSX (37.0%, 35.8%, and 4.5%, respectively). Patients with obesity or cardiovascular history before transplantation received more ATG than BSX, as were patients who received a graft from a marginal donor or with prolonged CIT. We also observed that patients receiving a preemptive transplant received more BSX induction (20.5%) than ATG (14.4%).

**Table 2:** Cohort characteristics according to induction therapy.

	NA	Overall (n = 4157)		ATG (n = 2195)		BSX (n = 1962)		p-value
**Recipient characteristics**								
Recipient age (years)	0	53.1	(14.8)	53.6	(14.4)	52.5	(15.3)	0.011
Male recipient	0	2670	(64.2)	1314	(59.9)	1356	(69.1)	<0.001
Recipient BMI ≥ 30 kg/m^2^	24	649	(15.7)	373	(17.1)	276	(14.1)	0.008
Diabetes history	0	787	(18.9)	437	(19.9)	350	(17.8)	0.089
Cardiovascular history (*)	0	1584	(38.1)	925	(42.1)	659	(33.6)	<0.001
Cancer history	0	545	(13.1)	292	(13.3)	253	(12.9)	0.697
Positive CMV	12	2605	(62.8)	1433	(65.5)	1172	(59.9)	<0.001
Detectable anti-HLA class I	0	1985	(47.8)	1259	(57.4)	726	(37.0)	<0.001
Detectable anti-HLA class II	0	1908	(45.9)	1206	(54.9)	702	(35.8)	<0.001
Renal replacement therapy	15							<0.001
Preemptive transplant		717	(17.3)	316	(14.4)	401	(20.5)	
Peritoneal dialysis		395	(9.5)	184	(8.4)	211	(10.8)	
Hemodialysis		3030	(73.2)	1687	(77.1)	1343	(68.7)	
**Donor characteristics**								
Donor age (years)	21	55.3	(16.5)	56.0	(16.0)	54.6	(17.0)	0.005
Male donor	3	2178	(52.4)	1199	(54.7)	979	(49.9)	0.002
Living donor	0	904	(21.7)	350	(15.9)	554	(28.2)	<0.001
Positive CMV	0	2347	(56.5)	1225	(55.8)	1122	(57.2)	0.371
EBV mismatch (+/-)	10	137	(3.3)	44	(2.0)	93	(4.7)	<0.001
**Graft characteristics**								
Year	0							0.003
2013 to 2015		1839	(44.2)	945	(43.1)	894	(45.6)	
2016–2017		1294	(31.1)	662	(30.2)	632	(32.2)	
2018–2019		1024	(24.6)	588	(26.8)	436	(22.2)	
Re-transplantation	0	598	(14.4)	509	(23.2)	89	(4.5)	<0.001
Last donor creat. ≥ 132.6 μmol/L	12	431	(10.4)	290	(13.2)	141	(7.2)	<0.001
HLA incompatibilities > 4	55	717	(17.5)	360	(16.7)	357	(18.3)	0.184
Cold ischemia time (hours)	43	13.7	(8.4)	14.9	(8.2)	12.4	(8.5)	<0.001

Abbreviations: ATG, anti-thymocyte globulin; BMI, body mass index; BSX, Basiliximab; CMV, cytomegalovirus; CMV R+, CMV seropositive recipient; CMV D+, CMV seropositive donor; EBV, Epstein-Barr virus; EBV D+R-, EBV seronegative recipient from EBV-seropositive donor; HLA, human leucocyte antigen NA, number of missing values. Continuous characteristics are presented as means (standard deviation). The qualitative values are presented as the effective (n) modality followed by its percentage. (*) Excluding hypertension. (+/-) EBV positive in the donor and negative in the recipient.

## Decision of induction therapy

We aimed to more explicitly describe the decision of induction therapy. We did not consider several subgroups in the models’ estimations because of the corresponding quasi-systematic ATG decision: second kidney transplants in all centers except E, recipients with detectable anti-HLA class I immunization in center B, and transplantations from donors with a last creatinemia higher than 132.6 μmol/L in centers C and D.

We retained fifteen factors significantly associated with the induction prescription in at least one center: recipient age, gender, BMI, cardiovascular history, cancer history, recipient CMV status, pre-transplantation anti-HLA immunization against class I and II, renal replacement therapy, donor type, donor CMV status, year and rank of transplantation, last donor creatinemia, and CIT. We did not retain the EBV serostatus because too few seronegative recipients received a transplant from a seropositive donor.

**Fig 2:**
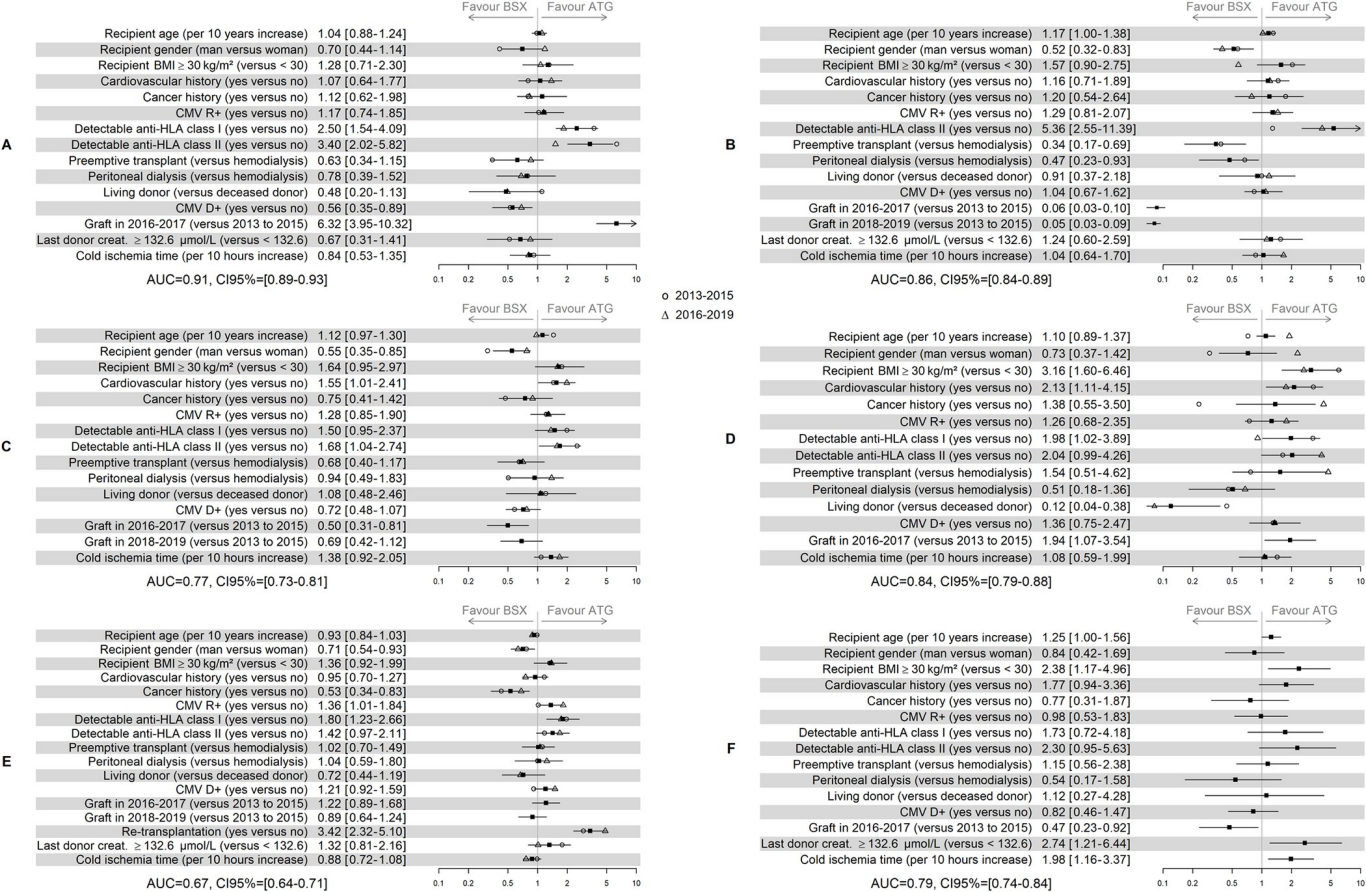
Results of the six logistic regressions aiming to illustrate the factors associated with the choice of induction therapy. n = 484 for center A (only first transplant and transplants performed from 2013 to 2017), n = 604 for B (only first transplant and non-detectable anti-HLA Class I), n = 577 for C (only first transplant and last donor creatinemia < 132.6 μmol/L), n = 276 for D (only first transplant and last donor creatinemia < 132.6 μmol/L), n = 1045 for E, and n = 271 for F (only first transplant).

Fig 2 shows the prescription decision process. An Odds-Ratio (OR) greater than 1 reflects increased ATG use. Favoring ATG as induction therapy was a homogeneous practice in re-transplanted patients and recipients with anti-HLA class I or class II immunization. In contrast, for the other predictive characteristics, we observed heterogeneities between centers. For instance, we observed significant differences between the different time-frames for the different centers; center A prescribed more ATG over time, while center B prescribed more BSX over time. From 2016 to 2017, centers C and F prescribed more BSX compared to 2013–2015. We observed the opposite trend for center D. Finally, no tendency to change was observed for the center E.

The AUC values ranged from 0.67 (CI95% from 0.64 to 0.71) to 0.91 (CI95% from 0.89 to 0.93). The higher the AUC value, the more that decision was predictable, i.e., the center protocol was standardized. Despite the wide variability in prescribing ATG between centers, these results illustrate that the centers seem to define their own (randomless) rules.

Fig 2 also reports the OR according to the timeframe (2013–2015 and 2016–2019). For these analyses, note that logistic regressions were conducted only for the hemodialyzed patients in center B for the period 2016–2019, and only for patients with a last donor creatininemia lower than 132.6 μmol/L for center D during the period from 2013 to 2015, ATG being used systematically in these populations. These analyses were not conducted for center F due to the small number of grafts in 2016. Independent of the timeframe, transplantation of a second graft was associated with greater ATG use compared to a first transplant. For the other characteristics, as in the main analysis, we observed heterogeneities between centers in each period. For example, recipient age was not associated with the choice of induction therapy in centers A and E, regardless of the timeframe. For centers B and C, older patients received more ATG during the period from 2013 to 2015 compared to 2016–2019. Finally, BSX was prescribed more for older patients in center D from 2013 to 2015 whereas ATG was higher from 2016 to 2019.

## Standardized results to improve inter-center comparisons

We previously reported that ATG center-specific prevalence ranged from 41% to 75%. This range may be partially explained by the differences in the patient and transplantation characteristics between centers. By using the previous center-specific predictive models, we computed the expected (standardized) ATG prevalence in each center in the counterfactual world where all centers had patients with characteristics like the overall cohort (first column, Table 1). These standardized prevalence values were 58%, 71%, 74%, 57%, 34% and 48% for centers A to F, respectively.

We also computed the expected probability of receiving ATG in each center for two illustrative recipients by using the previous center-specific predictive models. The first recipient was a 35-year-old male, with a BMI < 30 kg/m^2^, without cardiovascular or cancer history, negative for CMV, without anti-HLA class I or II immunization, who received a first transplant after hemodialysis from a deceased donor, negative for CMV, with a last creatinemia < 132.6 μmol/L and a CIT equal to 28 hours. His expected probability of receiving ATG was 36.6%, 5.5%, 48.2%, 26.3%, 17.5% and 21.7% from centers A to F, respectively. For a similar patient with anti-HLA class I immunization before transplantation, the probabilities were 59.1%, 100.0%, 58.2%, 41.4%, 27.6%, and 32.5% from centers A to F, respectively.

## Discussion

In this study, we describe for the first time the choice between ATG and BSX and its evolution as induction therapy in a multicentric French cohort of kidney transplant recipients. Approximately half of the patients received ATG, and we noted that there was considerable heterogeneity between centers (ranging from 41% to 75%). We also found that the factors associated with this decision were highly variable between centers, and we quantified this variabilty. Whilst it was not systematic, all centers tended to respect the international guidelines and preferred ATG induction therapy for all re-transplanted patients or almost all immunological high-risk patients with anti-HLA class I or class II immunization [20, 21, 25]. Considering the overall picture, most situations known to be associated with a high risk of delayed graft function are more frequently managed with ATG. Nevertheless, with the exception of anti-HLA immunization, we found that patient and transplantation characteristics associated with the decision to use ATG was highly variable between centers. More specifically, this did not appear to be guided by standardized rules but seemed to depend more on center-specific practices than on evidence-based results, as was shown for US transplant centers [22].

This raises the question of whether the current guidelines relating to the definition of the immunological risk of patients should be updated, given that these guidelines were drafted in an era before the current sensitive methods of anti-HLA antibody detection, *i*.*e*., Luminex assay (that is used in 95% of patients in our cohort). Moreover, monitoring of additional epitope mismatches, associated with increased allograft rejection risk [26], will also need to be included shortly [27, 28]. Overall, new data from emerging technologies that provide a better understanding of the pathophysiological processes must be incorporated to precisely define the pre-transplant risk of rejection and, therefore, the necessity for induction therapy by T-cell depletion.

Our results, obtained from analysis of a contempory European cohort, are in accordance with older studies conducted in the US [22–24]. This illustrates that despite efforts to standardize practices in the past, these remain heterogeneous. For instance, three of the French centers we studied did not change their medical decision making practices regarding the patient age. In contrast, two other centers changed their age-related ATG administration practices, but in opposite directions.

Clinicians need strong recommendations for prescribing induction therapy, particularly for low immunological risk patients, to avoid arbitrary decisions of the medical staff [20]. Whilst improved evidence based medicine may in general be supported by the weight of the literature, it is crucial to carefully filter and re-assess data using meta-analysis stratified by patient profiles.

Besides the lack of standard rules for induction decision-making, it was nevertheless interesting to observe that several centers referred to specific protocols that are highly standardized (AUC > 0.85 for two centers). Moreover, the specific center protocol is all the more possible in France because the social security system reimburses induction treatment on the basis of their authorization for use and not on the basis of a cost system.

It would have been interesting to assess the long-term results of such practices of induction therapy, but such a study is beyond our objectives. Our aim was not to find the best therapeutic option for patients but to underline the variability of practices between centers.

As usual in observational studies, our study presents several limitations. First, we excluded 260 grafts due to missing anti-HLA class I or II data. We observed more missing data for grafts performed in-between 2016 and 2017 and for re-transplantation. Second, because of the absence of systematic collection practices, the history of DSA was not considered in our analyses. Third, there were different thresholds between centers regarding the definition of positive anti-HLA class I and II, which can partially explain the differences in ATG prescription. However, we reported that standardized ATG prevalence values varied from 34% to 74%, supporting the fact that immunization, and patient profiles more generally, did not seem to explain practice heterogeneity. Note also that we did not have the details of the commercial kits used to detect anti-HLA immunization. Fourth, we were not able to account for research protocols that imposed the choice of induction therapy, as we did not collect this information in our database. Finally, we did not consider the ATG dose, which is also not collected. Recent pilot studies have reported the potential benefit of reduced ATG duration and dosage with good tolerance [29, 30]. Whilst this may have influenced the induction strategy in some centers, to date, a randomized trial to compare different dosage strategies has not been conducted.

In conclusion, our study quantified the persistant high variability of current practices in the choice of induction therapy, except for patients with high immunological risk. Our results call for more consensus, clinical research with more persuasive evidence, and meta-analysis stratified by patient profiles, especially for kidney transplant recipients with low immunological risk.

## Supporting information

S1 Table Comparison of the included versus excluded grafts because of missing data on anti-HLA class I or anti-HLA class II. (DOCX)

S2 Table Characteristics at transplantation according to the induction therapy in center A. (DOCX)

S3 Table Characteristics at transplantation according to the induction therapy in center B. (DOCX)

S4 Table Characteristics at transplantation according to the induction therapy in center C. (DOCX)

S5 Table Characteristics at transplantation according to the induction therapy in center D. (DOCX)

S6 Table Characteristics at transplantation according to the induction therapy in center E. (DOCX)

S7 Table Characteristics at transplantation according to the induction therapy in center F. (DOCX)

S1 Data (XLSX)
