# Supplementary material for: Induction therapy in kidney transplant recipients: Description of the practices according to the calendar period from the French multicentric DIVAT cohort
Source: PLoS One. 2020 Oct 22;15(10):e0240929. doi: 10.1371/journal.pone.0240929 (PMC7580969; doi:10.1371/journal.pone.0240929)
Supplement: S1 Table — (DOCX) [file pone.0240929.s001.docx]

**S1 Table.** Comparison of the included versus excluded grafts because of missing data on anti-HLA class I or anti-HLA class II

|  | **NA** | **Included (n=4157)** | | **Excluded (n=260)** | | **p-value** |
| --- | --- | --- | --- | --- | --- | --- |
| **Recipient characteristics** |  |  |  |  |  |  |
| Recipient age (years) | 0 | 53.1 | (14.8) | 52.1 | (14.4) | <0.001 |
| Male recipients | 0 | 2670 | (64.2) | 177 | (68.1) | 0.209 |
| Recipient BMI ≥ 30 kg/m² | 26 | 649 | (15.7) | 41 | (15.9) | 0.936 |
| Diabetes history | 0 | 787 | (18.9) | 48 | (18.5) | 0.851 |
| Cardiovascular history (*) | 0 | 1584 | (38.1) | 104 | (40.0) | 0.542 |
| Cancer history | 0 | 545 | (13.1) | 39 | (15.0) | 0.383 |
| CMV R+ | 12 | 2605 | (62.8) | 164 | (63.1) | 0.941 |
| Renal replacement therapy | 18 |  |  |  |  | 0.121 |
| Preemptive transplant |  | 717 | (17.3) | 32 | (12.5) |  |
| Peritoneal dialysis |  | 395 | (9.5) | 28 | (10.9) |  |
| Hemodialysis |  | 3030 | (73.2) | 197 | (76.7) |  |
| **Donor characteristics** |  |  |  |  |  |  |
| Donor age (years) | 22 | 55.3 | (16.5) | 54.0 | (16.3) | <0.001 |
| Male donor | 3 | 2178 | (52.4) | 135 | (51.9) | 0.874 |
| Living donor | 0 | 904 | (21.7) | 47 | (18.1) | 0.163 |
| CMV D+ | 0 | 2347 | (56.5) | 134 | (51.5) | 0.121 |
| EBV mismatch (+/-) | 10 | 137 | (3.3) | 8 | (3.1) | 0.842 |
| **Graft characteristics** |  |  |  |  |  |  |
| Year | 0 |  |  |  |  | <0.001 |
| 2013 to 2015 |  | 1839 | (44.2) | 120 | (46.2) |  |
| 2016 – 2017 |  | 1294 | (31.1) | 108 | (41.5) |  |
| 2018 – 2019 |  | 1024 | (24.6) | 32 | (12.3) |  |
| Re-transplantation | 0 | 598 | (14.4) | 59 | (22.7) | <0.001 |
| Last donor creat. ≥ 132.6 µmol/L | 14 | 431 | (10.4) | 25 | (9.7) | 0.717 |
| HLA incompatibilities > 4 | 62 | 717 | (17.5) | 45 | (17.8) | 0.901 |
| Cold ischemia time (hours) | 49 | 13.7 | (8.4) | 14.6 | (7.9) | <0.001 |
| ATG as induction therapy | 0 | 2195 | (52.8) | 152 | (58.5) | 0.076 |

^Abbreviations: ATG, Anti-Thymocyte Globulin; BMI, body mass index; CMV R+, CMV seropositive recipient; CMV D+, CMV seropositive donor; EBV, Epstein-Barr virus; HLA, human leucocyte antigen; NA, number of missing values. Continuous characteristics are presented as means (standard deviation). The qualitative values are presented as the effective (n) modality followed by its percentage. (*) Excluding hypertension. (+/-) EBV positive in the donor and negative in the recipient.^
