# Supplementary material for: Induction therapy in kidney transplant recipients: Description of the practices according to the calendar period from the French multicentric DIVAT cohort
Source: PLoS One. 2020 Oct 22;15(10):e0240929. doi: 10.1371/journal.pone.0240929 (PMC7580969; doi:10.1371/journal.pone.0240929)
Supplement: S2 Table — (DOCX) [file pone.0240929.s002.docx]

**S2 Table.** Characteristics at transplantation according to the induction therapy in center A.

| **Center A** | **NA** | **ATG**  **(n=468)** | | **BSX**  **(n=344)** | | **p-value** |
| --- | --- | --- | --- | --- | --- | --- |
| **Recipient characteristics** |  |  |  |  |  |  |
| Recipient age (years) | 0 | 55.5 | (14.8) | 54.9 | (15.0) | 0.562 |
| Male recipient | 0 | 280 | (59.8) | 221 | (64.2) | 0.201 |
| Recipient BMI ≥ 30 kg/m² | 0 | 63 | (13.5) | 63 | (18.3) | 0.059 |
| Diabetes history | 0 | 97 | (20.7) | 54 | (15.7) | 0.069 |
| Cardiovascular history (*) | 0 | 241 | (51.5) | 132 | (38.4) | < 0.001 |
| Cancer history | 0 | 101 | (21.6) | 59 | (17.2) | 0.117 |
| CMV R+ | 1 | 231 | (49.5) | 160 | (46.5) | 0.406 |
| Detectable anti-HLA class I | 0 | 230 | (49.1) | 78 | (22.7) | < 0.001 |
| Detectable anti-HLA class II | 0 | 205 | (43.8) | 54 | (15.7) | < 0.001 |
| Renal replacement therapy | 0 |  |  |  |  | 0.012 |
| Preemptive transplant |  | 87 | (18.6) | 86 | (25.0) |  |
| Peritoneal dialysis |  | 50 | (10.7) | 49 | (14.2) |  |
| Hemodialysis |  | 331 | (70.7) | 209 | (60.8) |  |
| **Donor characteristics** |  |  |  |  |  |  |
| Donor age (years) | 1 | 58.0 | (15.8) | 56.5 | (16.3) | 0.208 |
| Male donor | 0 | 255 | (54.5) | 183 | (53.2) | 0.716 |
| Living donor | 0 | 84 | (17.9) | 93 | (27.0) | 0.002 |
| CMV D+ | 0 | 202 | (43.2) | 170 | (49.4) | 0.077 |
| EBV mismatch (+/-) | 1 | 11 | (2.4) | 23 | (6.7) | 0.002 |
| **Graft characteristics** |  |  |  |  |  |  |
| Year | 0 |  |  |  |  | 0.074 |
| 2013 to 2015 |  | 113 | (24.2) | 227 | (66.0) |  |
| 2016 – 2017 |  | 155 | (33.1) | 103 | (29.9) |  |
| 2018 – 2019 |  | **200** | **(42.7)** | **14** | **(4.1)** |  |
| Re-transplantation | 0 | 146 | (31.2) | 3 | (0.9) | < 0.001 |
| Last donor creat. ≥ 132.6 µmol/L | 0 | 50 | (10.7) | 37 | (10.8) | 0.974 |
| HLA incompatibilities > 4 | 0 | 108 | (23.1) | 78 | (22.7) | 0.893 |
| Cold ischemia time (hours) | 0 | 12.5 | (7.4) | 11.3 | (7.5) | 0.028 |

^Abbreviations: ATG, Anti-Thymocyte Globulin; BMI, body mass index; BSX, Basiliximab; CMV, cytomegalovirus; CMV R+, CMV seropositive recipient; CMV D+, CMV seropositive donor; EBV, Epstein-Barr virus; NA: number of missing values. Continuous characteristics are presented as means (standard deviation). The qualitative values are presented as the effective (n) modality followed by its percentage. (*) Excluding hypertension. (+/-) EBV positive in the donor and negative in the recipient.^
