# Supplementary material for: Induction therapy in kidney transplant recipients: Description of the practices according to the calendar period from the French multicentric DIVAT cohort
Source: PLoS One. 2020 Oct 22;15(10):e0240929. doi: 10.1371/journal.pone.0240929 (PMC7580969; doi:10.1371/journal.pone.0240929)
Supplement: S3 Table — (DOCX) [file pone.0240929.s003.docx]

**S3 Table.** Characteristics at transplantation according to the induction therapy in center B.

| **Center B** | **NA** | **ATG**  **(n=360)** | | **BSX**  **(n=449)** | | **p-value** |
| --- | --- | --- | --- | --- | --- | --- |
| **Recipient characteristics** |  |  |  |  |  |  |
| Recipient age (years) | 0 | 52.5 | (13.7) | 50.8 | (15.4) | 0.087 |
| Male recipient | 0 | 184 | (51.1) | 308 | (68.6) | < 0.001 |
| Recipient BMI ≥ 30 kg/m² | 0 | 67 | (18.6) | 69 | (15.4) | 0.220 |
| Diabetes history | 0 | 63 | (17.5) | 81 | (18.0) | 0.842 |
| Cardiovascular history ^a^ | 0 | 113 | (31.4) | 122 | (27.2) | 0.189 |
| Cancer history | 0 | 36 | (10.0) | 36 | (8.0) | 0.325 |
| CMV R+ | 1 | 255 | (71.0) | 287 | (63.9) | 0.033 |
| Detectable anti-HLA class I | 0 | **128** | **(35.6)** | **26** | **(5.8)** | < 0.001 |
| Detectable anti-HLA class II | 0 | 130 | (36.1) | 38 | (8.5) | < 0.001 |
| Renal replacement therapy | 0 |  |  |  |  | 0.001 |
| Preemptive transplant |  | 32 | (8.9) | 73 | (16.3) |  |
| Peritoneal dialysis |  | 35 | (9.7) | 61 | (13.6) |  |
| Hemodialysis |  | 293 | (81.4) | 315 | (70.1) |  |
| **Donor characteristics** |  |  |  |  |  |  |
| Donor age (years) | 0 | 55.1 | (16.5) | 52.7 | (18.6) | 0.052 |
| Male donor | 0 | 211 | (58.6) | 241 | (53.7) | 0.160 |
| Living donor | 0 | 40 | (11.1) | 85 | (18.9) | 0.002 |
| CMV D+ | 0 | 205 | (56.9) | 258 | (57.5) | 0.883 |
| EBV mismatch (+/-) | 0 | 6 | (1.7) | 20 | (4.5) | 0.025 |
| **Graft characteristics** |  |  |  |  |  |  |
| Year | 0 |  |  |  |  | < 0.001 |
| 2013 to 2015 |  | 221 | (61.4) | 90 | (20.0) |  |
| 2016 – 2017 |  | 74 | (20.6) | 186 | (41.5) |  |
| 2018 – 2019 |  | 65 | (18.0) | 173 | (38.5) |  |
| Re-transplantation | 0 | **86** | **(23.9)** | **14** | **(3.1)** | < 0.001 |
| Last donor creat. ≥ 132.6 µmol/L | 0 | 46 | (12.8) | 34 | (7.6) | 0.014 |
| HLA incompatibilities > 4 | 2 | 39 | (10.8) | 85 | (19.0) | 0.001 |
| Cold ischemia time (hours) | 4 | 13.6 | (6.5) | 10.8 | (6.4) | < 0.001 |

^Abbreviations: ATG, Anti-Thymocyte Globulin; BMI, body mass index; BSX, Basiliximab; CMV, cytomegalovirus; CMV R+, CMV seropositive recipient; CMV D+, CMV seropositive donor; EBV, Epstein-Barr virus; NA: number of missing values. Continuous characteristics are presented as means (standard deviation). The qualitative values are presented as the effective (n) modality followed by its percentage. (*) Excluding hypertension. (+/-) EBV positive in the donor and negative in the recipient.^
