# Supplementary material for: Induction therapy in kidney transplant recipients: Description of the practices according to the calendar period from the French multicentric DIVAT cohort
Source: PLoS One. 2020 Oct 22;15(10):e0240929. doi: 10.1371/journal.pone.0240929 (PMC7580969; doi:10.1371/journal.pone.0240929)
Supplement: S4 Table — (DOCX) [file pone.0240929.s004.docx]

**S4 Table.** Characteristics at transplantation according to the induction therapy in center C.

| **Center C** | **NA** | **ATG**  **(n=611)** | | **BSX**  **(n=206)** | | **p-value** |
| --- | --- | --- | --- | --- | --- | --- |
| **Recipient characteristics** |  |  |  |  |  |  |
| Recipient age (years) | 0 | 54.9 | (13.6) | 52.2 | (14.5) | 0.020 |
| Male recipient | 0 | 383 | (62.7) | 163 | (79.1) | < 0.001 |
| Recipient BMI ≥ 30 kg/m² | 22 | 99 | (16.7) | 19 | (9.5) | 0.013 |
| Diabetes history | 0 | 122 | (20.0) | 29 | (14.1) | 0.060 |
| Cardiovascular history ^a^ | 0 | 245 | (40.1) | 58 | (28.2) | 0.002 |
| Cancer history | 0 | 74 | (12.1) | 26 | (12.6) | 0.847 |
| CMV R+ | 5 | 407 | (67.2) | 120 | (58.3) | 0.021 |
| Detectable anti-HLA class I | 0 | 393 | (64.3) | 81 | (39.3) | < 0.001 |
| Detectable anti-HLA class II | 0 | 368 | (60.2) | 74 | (35.9) | < 0.001 |
| Renal replacement therapy | 12 |  |  |  |  | 0.004 |
| Preemptive transplant |  | 77 | (12.8) | 44 | (21.8) |  |
| Peritoneal dialysis |  | 48 | (7.9) | 20 | (9.9) |  |
| Hemodialysis |  | 478 | (79.3) | 138 | (68.3) |  |
| **Donor characteristics** |  |  |  |  |  |  |
| Donor age (years) | 16 | 56.3 | (16.3) | 55.4 | (16.9) | 0.535 |
| Male donor | 1 | 333 | (54.6) | 97 | (47.1) | 0.062 |
| Living donor | 0 | 70 | (11.5) | 60 | (29.1) | < 0.001 |
| CMV D+ | 0 | 362 | (59.2) | 133 | (64.6) | 0.177 |
| EBV mismatch (+/-) | 5 | 7 | (1.2) | 5 | (2.4) | 0.193 |
| **Graft characteristics** |  |  |  |  |  |  |
| Year | 0 |  |  |  |  | 0.100 |
| 2013 to 2015 |  | 244 | (40.0) | 83 | (40.3) |  |
| 2016 – 2017 |  | 159 | (26.0) | 67 | (32.5) |  |
| 2018 – 2019 |  | 208 | (34.0) | 56 | (27.2) |  |
| Re-transplantation | 0 | **106** | **(17.3)** | **4** | **(1.9)** | < 0.001 |
| Last donor creat. ≥ 132.6 µmol/L | 4 | **83** | **(13.7)** | **5** | **(2.4)** | < 0.001 |
| HLA incompatibilities > 4 | 51 | 82 | (14.4) | 37 | (18.9) | 0.134 |
| Cold ischemia time (hours) | 24 | 17.0 | (7.6) | 13.0 | (7.9) | < 0.001 |

^Abbreviations: ATG, Anti-Thymocyte Globulin; BMI, body mass index; BSX, Basiliximab; CMV, cytomegalovirus; CMV R+, CMV seropositive recipient; CMV D+, CMV seropositive donor; EBV, Epstein-Barr virus; NA: number of missing values. Continuous characteristics are presented as means (standard deviation). The qualitative values are presented as the effective (n) modality followed by its percentage. (*) Excluding hypertension. (+/-) EBV positive in the donor and negative in the recipient.^
