# Supplementary material for: Induction therapy in kidney transplant recipients: Description of the practices according to the calendar period from the French multicentric DIVAT cohort
Source: PLoS One. 2020 Oct 22;15(10):e0240929. doi: 10.1371/journal.pone.0240929 (PMC7580969; doi:10.1371/journal.pone.0240929)
Supplement: S5 Table — (DOCX) [file pone.0240929.s005.docx]

**S5 Table.** Characteristics at transplantation according to the induction therapy in center D.

| **Center D** | **NA** | **ATG**  **(n=185)** | | **BSX**  **(n=175)** | | **p-value** |
| --- | --- | --- | --- | --- | --- | --- |
| **Recipient characteristics** |  |  |  |  |  |  |
| Recipient age (years) | 0 | 54.0 | (15.1) | 52.0 | (15.9) | 0.220 |
| Male recipient | 0 | 123 | (66.5) | 124 | (70.9) | 0.372 |
| Recipient BMI ≥ 30 kg/m² | 0 | 45 | (24.3) | 27 | (15.4) | 0.035 |
| Diabetes history | 0 | 42 | (22.7) | 30 | (17.1) | 0.187 |
| Cardiovascular history ^a^ | 0 | 127 | (68.6) | 83 | (47.4) | < 0.001 |
| Cancer history | 0 | 22 | (11.9) | 19 | (10.9) | 0.757 |
| CMV R+ | 0 | 119 | (64.3) | 93 | (53.1) | 0.031 |
| Detectable anti-HLA class I | 0 | 82 | (44.3) | 40 | (22.9) | < 0.001 |
| Detectable anti-HLA class II | 0 | 78 | (42.2) | 28 | (16.0) | < 0.001 |
| Renal replacement therapy | 0 |  |  |  |  | 0.022 |
| Preemptive transplant |  | 16 | (8.7) | 30 | (17.1) |  |
| Peritoneal dialysis |  | 18 | (9.7) | 23 | (13.2) |  |
| Hemodialysis |  | 151 | (81.6) | 122 | (69.7) |  |
| **Donor characteristics** |  |  |  |  |  |  |
| Donor age (years) | 0 | 54.9 | (16.6) | 51.7 | (16.5) | 0.072 |
| Male donor | 0 | 102 | (55.1) | 74 | (42.3) | 0.015 |
| Living donor | 0 | 21 | (11.4) | 82 | (46.9) | < 0.001 |
| CMV D+ | 0 | 105 | (56.8) | 97 | (55.4) | 0.800 |
| EBV mismatch (+/-) | 0 | 6 | (3.2) | 4 | (2.3) | 0.751 |
| **Graft characteristics** |  |  |  |  |  |  |
| Year | 0 |  |  |  |  | < 0.001 |
| 2013 to 2015 |  | 76 | (41.1) | 111 | (63.4) |  |
| 2016 – 2017 |  | 109 | (58.9) | 64 | (36.6) |  |
| Re-transplantation | 0 | **35** | **(18.9)** | **8** | **(4.6)** | < 0.001 |
| Last donor creat. ≥ 132.6 µmol/L | 2 | **36** | **(19.5)** | **6** | **(3.5)** | < 0.001 |
| HLA incompatibilities > 4 | 1 | 31 | (16.8) | 25 | (14.3) | 0.504 |
| Cold ischemia time (hours) | 2 | 15.9 | (7.0) | 10.8 | (7.7) | < 0.001 |

^Abbreviations: ATG, Anti-Thymocyte Globulin; BMI, body mass index; BSX, Basiliximab; CMV, cytomegalovirus; CMV R+, CMV seropositive recipient; CMV D+, CMV seropositive donor; EBV, Epstein-Barr virus; NA, number of missing values. Continuous characteristics are presented as means (standard deviation). The qualitative values are presented as the effective (n) modality followed by its percentage. (*) Excluding hypertension. (+/-) EBV positive in the donor and negative in the recipient.^
