# Supplementary material for: Induction therapy in kidney transplant recipients: Description of the practices according to the calendar period from the French multicentric DIVAT cohort
Source: PLoS One. 2020 Oct 22;15(10):e0240929. doi: 10.1371/journal.pone.0240929 (PMC7580969; doi:10.1371/journal.pone.0240929)
Supplement: S6 Table — (DOCX) [file pone.0240929.s006.docx]

**S6 Table.** Characteristics at transplantation according to the induction therapy in center E.

| **Center E** | **NA** | **ATG**  **(n=428)** | | **BSX**  **(n=617)** | | **p-value** |
| --- | --- | --- | --- | --- | --- | --- |
| **Recipient characteristics** |  |  |  |  |  |  |
| Recipient age (years) | 0 | 48.8 | (14.1) | 51.8 | (15.2) | 0.001 |
| Male recipient | 0 | 248 | (57.9) | 411 | (66.6) | 0.004 |
| Recipient BMI ≥ 30 kg/m² | 2 | 65 | (15.2) | 77 | (12.5) | 0.207 |
| Diabetes history | 0 | 73 | (17.1) | 120 | (19.4) | 0.327 |
| Cardiovascular history ^a^ | 0 | 151 | (35.3) | 223 | (36.1) | 0.775 |
| Cancer history | 0 | 40 | (9.3) | 90 | (14.6) | 0.012 |
| CMV R+ | 5 | 315 | (73.6) | 400 | (65.4) | 0.005 |
| Detectable anti-HLA class I | 0 | 378 | (88.3) | 478 | (77.5) | < 0.001 |
| Detectable anti-HLA class II | 0 | 376 | (87.9) | 490 | (79.4) | < 0.001 |
| Renal replacement therapy | 2 |  |  |  |  | 0.444 |
| Preemptive transplant |  | 77 | (18.0) | 130 | (21.1) |  |
| Peritoneal dialysis |  | 26 | (6.1) | 38 | (6.2) |  |
| Hemodialysis |  | 325 | (75.9) | 447 | (72.7) |  |
| **Donor characteristics** |  |  |  |  |  |  |
| Donor age (years) | 2 | 52.9 | (14.8) | 54.6 | (16.3) | 0.088 |
| Male donor | 1 | 225 | (52.7) | 305 | (49.4) | 0.300 |
| Living donor | 0 | 126 | (29.4) | 208 | (33.7) | 0.145 |
| CMV D+ | 0 | 263 | (61.4) | 358 | (58.0) | 0.267 |
| EBV mismatch (+/-) | 4 | 12 | (2.8) | 40 | (6.5) | 0.007 |
| **Graft characteristics** |  |  |  |  |  |  |
| Year | 0 |  |  |  |  | 0.112 |
| 2013 to 2015 |  | 178 | (41.6) | 263 | (42.6) |  |
| 2016 – 2017 |  | 135 | (31.5) | 161 | (26.1) |  |
| 2018 – 2019 |  | 115 | (26.9) | 193 | (31.3) |  |
| Re-transplantation | 0 | 104 | (24.3) | 54 | (8.8) | < 0.001 |
| Last donor creat. ≥ 132.6 µmol/L | 5 | 41 | (9.6) | 44 | (7.2) | 0.155 |
| HLA incompatibilities > 4 | 1 | 79 | (18.5) | 104 | (16.9) | 0.492 |
| Cold ischemia time (hours) | 10 | 14.5 | (10.6) | 13.9 | (10.7) | 0.381 |

^Abbreviations: ATG, Anti-Thymocyte Globulin; BMI, body mass index; BSX, Basiliximab; CMV, cytomegalovirus; CMV R+, CMV seropositive recipient; CMV D+, CMV seropositive donor; EBV, Epstein-Barr virus; NA, number of missing values. Continuous characteristics are presented as means (standard deviation). The qualitative values are presented as the effective (n) modality followed by its percentage. (*) Excluding hypertension. (+/-) EBV positive in the donor and negative in the recipient.^
