# Supplementary material for: Induction therapy in kidney transplant recipients: Description of the practices according to the calendar period from the French multicentric DIVAT cohort
Source: PLoS One. 2020 Oct 22;15(10):e0240929. doi: 10.1371/journal.pone.0240929 (PMC7580969; doi:10.1371/journal.pone.0240929)
Supplement: S7 Table — (DOCX) [file pone.0240929.s007.docx]

**S7 Table.** Characteristics at transplantation according to the induction therapy in center F.

| **Center F** | **NA** | **ATG**  **(n=143)** | | **BSX**  **(n=171)** | | **p-value** |
| --- | --- | --- | --- | --- | --- | --- |
| **Recipient characteristics** |  |  |  |  |  |  |
| Recipient age (years) | 0 | 58.9 | (13.3) | 55.2 | (15.1) | 0.022 |
| Male recipient | 0 | 96 | (67.1) | 129 | (75.4) | 0.104 |
| Recipient BMI ≥ 30 kg/m² | 0 | 34 | (23.8) | 21 | (12.3) | 0.008 |
| Diabetes history | 0 | 40 | (28.0) | 36 | (21.1) | 0.154 |
| Cardiovascular history ^a^ | 0 | 48 | (33.6) | 41 | (24.0) | 0.060 |
| Cancer history | 0 | 19 | (13.3) | 23 | (13.5) | 0.966 |
| CMV R+ | 0 | 106 | (74.1) | 112 | (65.5) | 0.098 |
| Detectable anti-HLA class I | 0 | 48 | (33.6) | 23 | (13.5) | <0.001 |
| Detectable anti-HLA class II | 0 | 49 | (34.3) | 18 | (10.5) | <0.001 |
| Renal replacement therapy | 1 |  |  |  |  | 0.053 |
| Preemptive transplant |  | 27 | (18.9) | 38 | (22.3) |  |
| Peritoneal dialysis |  | 7 | (4.9) | 20 | (11.8) |  |
| Hemodialysis |  | 109 | (76.2) | 112 | (65.9) |  |
| **Donor characteristics** |  |  |  |  |  |  |
| Donor age (years) | 2 | 61.9 | (14.4) | 57.5 | (16.0) | 0.011 |
| Male donor | 1 | 73 | (51.4) | 79 | (46.2) | 0.359 |
| Living donor | 0 | 9 | (6.3) | 26 | (15.2) | 0.012 |
| CMV D+ | 0 | 88 | (61.5) | 106 | (62.0) | 0.935 |
| EBV mismatch (+/-) | 0 | 2 | (1.4) | 1 | (0.6) | 0.593 |
| **Graft characteristics** |  |  |  |  |  |  |
| Year | 0 |  |  |  |  | 0.074 |
| 2013 to 2015 |  | 113 | (79.0) | 120 | (70.2) |  |
| 2016 – 2017 |  | 30 | (21.0) | 51 | (29.8) |  |
| Re-transplantation | 0 | **32** | **(22.4)** | **6** | **(3.5)** | <0.001 |
| Last donor creat. ≥ 132.6 µmol/L | 1 | 34 | (23.8) | 15 | (8.8) | <0.001 |
| HLA incompatibilities > 4 | 0 | 21 | (14.7) | 28 | (16.4) | 0.681 |
| Cold ischemia time (hours) | 3 | 17.7 | (7.0) | 14.2 | (6.8) | <0.001 |

^Abbreviations: ATG, Anti-Thymocyte Globulin; BMI, body mass index; BSX, Basiliximab; CMV, cytomegalovirus; CMV R+, CMV seropositive recipient; CMV D+, CMV seropositive donor; EBV, Epstein-Barr virus; NA, number of missing values. Continuous characteristics are presented as means (standard deviation). The qualitative values are presented as the effective (n) modality followed by its percentage. (*) Excluding hypertension. (+/-) EBV positive in the donor and negative in the recipient.^
